# Face masks have emotion-dependent dissociable effects on accuracy and confidence in identifying facial expressions of emotion

**DOI:** 10.1186/s41235-022-00366-w

**Published:** 2022-02-14

**Authors:** Emily Grenville, Dominic M. Dwyer

**Affiliations:** School of Psychology, Cardiff University, Tower Building, Park Place, Cardiff, CF10 3AT UK

**Keywords:** Facemask, Emotion recognition, Confidence, Accuracy, Bias

## Abstract

The coronavirus pandemic has resulted in increased use of face masks worldwide. Here, we examined the effect of wearing a face mask on the ability to recognise facial expressions of emotion. In a within-subjects design, 100 UK-based undergraduate students were shown facial expressions of anger, disgust, fear, happiness, sadness, and neutral expression; these were either posed with or without a face mask, or with a face mask artificially imposed onto them. Participants identified the emotion portrayed in the photographs from a fixed choice array of answers and rated their confidence in their selection. While overall accuracy was higher without than with masks, the effect varied across emotions, with a clear advantage without masks in disgust, happiness, and sadness; no effect for neutral, and lower accuracy without masks for anger and fear. In contrast, confidence was generally higher without masks, with the effect clear for all emotions other than anger. These results confirm that emotion recognition is affected by face mask wearing, but reveal that the effect depends on the emotion being displayed—with this emotion-dependence not reflected in subjects’ confidence. The disparity between the effects of mask wearing on different emotions and the failure of this to be reflected in confidence ratings suggests that mask wearing not only effects emotion recognition, but may also create biases in the perception of facial expressions of emotion of which perceivers are unaware. In addition, the similarity of results between the Imposed Mask and Posed Mask conditions suggests that prior research using artificially imposed masks has not been deleteriously affected by the use of this manipulation.

**Supplementary Information:**

The online version contains supplementary material available at 10.1186/s41235-022-00366-w.

## Introduction

Human faces provide valuable information about not only the identity of a person but also their emotional state (Bruce & Young, 1986). The functional utility of transmitting this information is well recognised (e.g. Ekman, 2003), as are the facts that emotion recognition in faces is both rapid (e.g. Tracy & Robins, 2008) and generally highly accurate (e.g. Ekman, 2003; Ekman & Friesen, 1971). Although there is debate over whether the expression and recognition of emotions is culturally universal (compare, Ekman, 1992; with, Jack et al., 2012).

Given the importance of the transmission and perception of emotions through facial expressions, the fact that one key protective action in the current COVID-19 pandemic is wearing of face masks (World Health Organisation, 2020) which obscure part of the face, raises the issue of the impact of mask wearing on emotion recognition in faces. Previous studies have shown that emotion recognition is impaired when only part of the face is visible (e.g. Bassili, 1979; Roberson et al., 2012) and so the natural presumption is that face mask wearing will also impair emotion recognition. Indeed, several recent studies have demonstrated exactly this effect (Carbon, 2020; Gori et al., 2021; Gulbetekin, 2021; Noyes et al., 2021; Pazhoohi et al., 2021). However, one potentially problematic feature of all these studies is that they deployed stimuli that were created by using image processing software to artificially impose a face mask, rather than use images of people expressing emotions while actually wearing a mask. The use of graphical manipulation does allow for the standardisation of the emotional expression between mask and no-mask conditions (Carbon, 2020); however, it also brings with it the possibility of stimulus artefacts due to the graphical manipulation itself that may impact on emotion recognition. Perhaps more importantly, it may be the case that some aspects of the contours of the lower part of the face that help demonstrate emotion may be discernible due to their effect on the shape of the mask itself: for example, the movement of the cheeks in a broad smile may raise the upper part of a mask or the opening of the mouth in surprise may stretch it vertically. Moreover, the facial expression of emotion may change when a person is wearing a mask (e.g. if they were to amplify, consciously or unconsciously, the emotion expression). Using image manipulation to impose masks negates the possibility of investigating such things in a naturalistic manner and may give an inaccurate assessment of the impacts of mask wearing on facial expression recognition.

While all studies of mask wearing effects on facial emotion recognition report generally deleterious effects of the mask on accuracy of recognition, the effects were not consistent across emotions. Gulbetekin (2021) and Pazhoohi et al. (2021) reported a reduction in accuracy for all emotions tested (although with different effect sizes across emotions); Carbon (2020) reported recognition deficits for angry, disgusted, happy, and sad, but not for fearful or neutral expressions; and Noyes et al. (2021) reported deficits for angry, disgusted, fearful, happy, and surprised, but not for sad or neutral expressions. Firstly, the fact that the effect of covering the mouth and lower face with masks differs across emotions is consistent with studies, using the “bubbles” (Gosselin & Schyns, 2001) or related methods, which suggest the most diagnostic areas of the face differ across emotions (e.g. Blais et al., 2012; Smith et al., 2005; Wegrzyn et al., 2017); and with studies of occlusion that have revealed differences between emotions in the effects of covering the eye vs mouth regions (e.g. Beaudry et al., 2014; Kotsia et al., 2008; Schurgin et al., 2014). Secondly, the fact that the heterogeneity of effect of masks across emotions was not consistent across studies is perhaps unsurprising given that prior studies of the importance of different face features/regions in different emotions themselves have produced somewhat inconsistent results. While from bubbles-based studies it appears that generally the mouth region is most informative for happy, surprised, and disgusted expressions, the eyes for fearful and angry expressions, and both mouth and eye regions for sad and neutral expressions (Blais et al., 2012; Smith et al., 2005; Wegrzyn et al., 2017), the only consistent result from comparing occlusion of the eye and mouth regions is that identification of happy expressions are more disrupted by mouth than eye occlusion, while other expressions have inconsistent effects: for example, Kotsia et al. (2008) found anger more disrupted by mouth than eye occlusion and disgust more disrupted by eye than mouth occlusion, while Schurgin et al. (2014) reported the opposite pattern of results.

In addition, only two of the previous studies of the effects of mask wearing on emotion recognition (Carbon, 2020; Pazhoohi et al., 2021) collected data on the confidence of the observers. In both studies, confidence was lower for all expressions when masks were present. Indeed, it is notable that the effect sizes for the effects of mask wearing on confidence were higher than for emotion recognition accuracy, and accuracy was degraded by masks only for some expressions in Carbon (2020). In Carbon’s original report, this difference between accuracy and confidence was not considered in any depth and largely dismissed as a being the product of ceiling effects obscuring accuracy differences in some emotions and nor was the difference in effect size for accuracy versus confidence discussed by Pazhoohi et al. (2021). However, if there is truly a discrepancy between the effects of masks on accuracy and confidence of emotion recognition, then it would suggest that observers do not have an accurate understanding of the degree to which their ability to determine emotional state from facial expression is impaired (or not). But whether there is a reliable dissociation between accuracy and confidence has yet to be confirmed because no other studies of mask wearing on emotion recognition collected confidence ratings.

Thus, in the current study we re-addressed the issue of the impact of face masks on the accuracy of emotion recognition, and directly compared graphically manipulated stimuli with stimuli where the emotions were posed by people wearing masks. In addition, we measured both accuracy of emotion recognition and confidence in those recognition judgements across different emotional expressions.

## Methods

### Participants

One-hundred psychology undergraduate students from Cardiff University were recruited for the study using the universities’ online experimental recruitment system. All participants received course credit for their participation. Participants were aged 18 to 38, with a mean age of 19.5 (SD 2.34): 9 were male and 91 were female, and participant-reported ethnicity was 80 white, 15 Asian, 1 black, and 4 mixed. All participants reported normal or corrected-to-normal vision. Participants in the study reported here provided informed consent and the research was approved by the Research Ethics Committee at Cardiff University (Title: Face processing: real and imagined. Ethics Code: EC.16.10.11.4606GA_EG).

### Materials

**Fig. 1:**
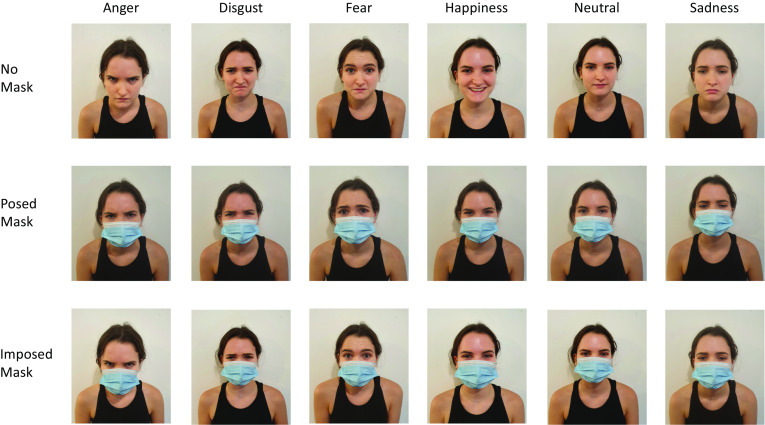
Stimulus examples. Shows stimulus examples from one person in the No Mask, Posed Mask, and Imposed Mask conditions for the six different emotions tested. The Imposed Mask condition stimuli were created by graphically imposing an image of a face mask over the images from the No Mask condition, while the Posed Mask condition stimuli were taken with the person expressing the relevant emotion while wearing a mask

A total of 108 images of six different people (not professional actors or models) expressing an emotion were used. All stimuli were posed by white females who wore black tops and were photographed, facing fully frontally and with the face and shoulders visible, by one of the researchers (EG) against a plain white background. Each person posed the expressions of anger, disgust, fear, happiness, sadness, and a neutral expression—once while wearing a face mask, and once without. The face masks used were disposable medical 3-Ply blue face masks that were fixed around the ears to cover the nose, mouth, cheeks, and chin. These images comprised the stimuli for the No Mask and Posed Mask conditions. The stimuli for the Imposed Mask condition were created by graphically imposing an image of the same type of disposable mask over the images posed without a face mask. There were thus six images of each emotion tested in each of the three mask conditions (see Fig. 1 for examples of the stimuli used).

### Design and procedure

The study was entirely within-subjects with the independent variables of mask condition (No Mask, Posed Mask, and Imposed Mask) and emotion (Anger, Disgust, Fear, happiness, Neutral, and Sadness). The dependent variables were the participants’ accuracy and confidence in identifying the emotions portrayed in the photographs.

The study was conducted online using Qualtrics software (Version April 2021, Qualtrics, Provo, UT). Following general information about the study and the provision of consent, participants providing responses to questions about their age, gender, and ethnicity. They were then informed that their task in the experiment was to classify the emotion being expressed in the images presented to them and indicate their confidence in this judgement. For each trial, a single image was presented and a fixed choice of 6 emotions (happiness, sadness, neutral, fearful, disgust and anger) was available below the image. Below these response options a cursor with a scale from 0 to 7 was presented to indicate how confident they were in their answer. One side of the scale (0) was labelled ‘very unconfident’, and the other side (7) was labelled ‘very confident’. There was no time limit per trial and the stimuli remained on screen until answers were provided for both the emotion displayed and confidence. The 108 trials were presented in random order. The experiment took approximately 20 min to complete.

### Data handling and analysis

Responses for the emotion displayed were categorised as correct or incorrect, and the number of correct responses (out of 6) per condition converted to a percentage for each participant. Confidence ratings were averaged across the 6 trials per condition for each participant. Both accuracy and confidence scores were analysed using repeated measures ANOVA with factors of mask condition and emotion (using Greenhouse–Geisser corrections where appropriate). Follow-up analyses of main effects and interactions were performed as t-tests. All analyses were performed using IMS SPSS Version 26. Because the subject pool was predominantly female and of self-reported while ethnicity, with low number of participants in other categories, it was not possible to perform a powerful analysis of participant gender or ethnicity. However, a re-analysis including only participants self-reported to be female and white revealed the same general pattern of results as reported below (this re-analysis is reported fully in the “Additional File 1” available with the online version of the paper).

## Results

**Fig. 2:**
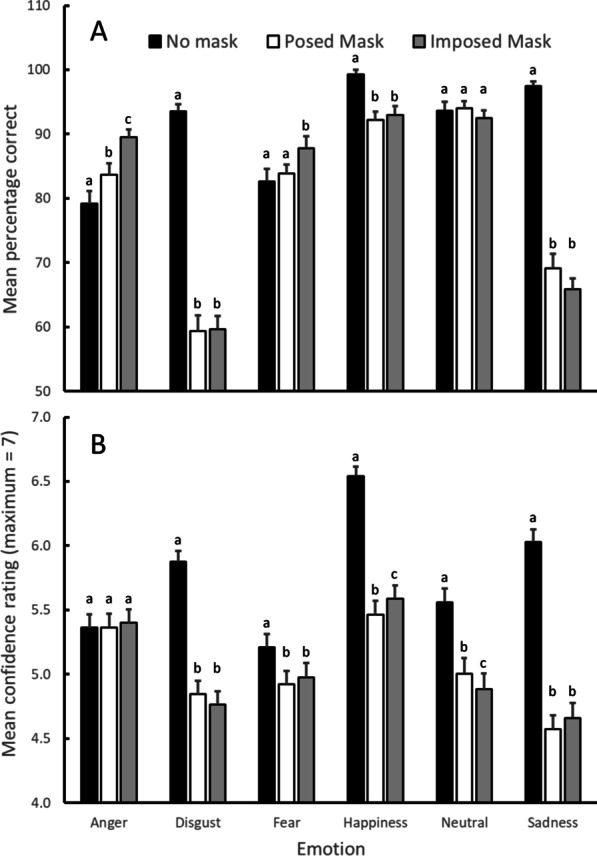
Accuracy and confidence results. **a** Mean percentage correct (with SEM) identification of emotional state, and **b** mean confidence rating (with SEM), as a function of emotion and mask condition. Note: superscript letters indicate the presence/absence of significant differences between mask conditions for each emotion: conditions with different letters are significantly different from each other (*p* < 0.05), conditions with the same letter are not significantly different (*p* > 0.05)

Inspection of Fig. 2a (showing mean percentage correct emotion identification across the six emotions and three mask conditions) suggests that overall accuracy varied across emotions, was generally better for the No Mask than the Posed Mask or Imposed Mask conditions, but that the effect of mask condition was not consistent across emotions (in particular, the advantage for the No Mask condition appears negligible or reversed for Anger, Fear, and Neutral emotions). These impressions are consistent with the results of the ANOVA analysis with significant effects of emotion [*F*(3.719, 368.2) = 69.197, *p* < 0.001, *η*^2^_*p*_ = 0.411], mask condition [*F*(2, 198) = 134.05, *p* < 0.001, *η*^2^_*p*_ = 0.575], and an interaction between emotion and mask condition [*F*(7.715, 763.8) = 63.388, *p* < 0.001, *η*^2^_*p*_ = 0.390]. Notwithstanding the mask condition by emotion interaction, it is potentially informative that follow-up tests of the main effect of mask condition revealed that accuracy was generally higher for the No Mask condition than either of the Posed Mask [*t*(99) = 13.99, *p* < 0.001] or Imposed Mask [*t*(99) = 13.21, *p* < 0.001] conditions, and that the two mask conditions were not significantly different from each other [*t*(99) = 1.56, *p* = 0.121].

Given the interaction between emotion and mask condition, follow-up tests were performed to compare the different mask conditions for each emotion separately. These revealed that for Anger accuracy was lower for the No Mask than either the Posed mask [*t*(99) = 2.48, *p* = 0.015] or Imposed Mask [*t*(99) = 5.46, *p* < 0.001] conditions, and that the two mask conditions were themselves significantly different [*t*(99) = 3.61, *p* < 0.001]; for Disgust accuracy was higher for the No Mask than either the Posed mask [*t*(99) = 13.81, *p* < 0.001] or Imposed Mask [*t*(99) = 15.04, *p* < 0.001] conditions, and that the two mask conditions were not significantly different [*t*(99) = 0.13, *p* = 0.899]; for Fear accuracy in the No Mask was not significantly different to the Posed mask condition [*t*(99) = 0.63, *p* = 0.527], but was lower than the Imposed Mask [*t*(99) = 2.62, *p* = 0.010] condition, and the two mask conditions were themselves significantly different [*t*(99) = 2.41, *p* = 0.018]; for Happiness accuracy was higher for the No Mask than either the Posed Mask [*t*(99) = 6.14, *p* < 0.001] or Imposed Mask [*t*(99) = 4.75, *p* < 0.001] conditions, and that the two mask conditions were not significantly different [*t*(99) = 0.75, *p* = 0.459]; for Neutral, there were no significant differences in accuracy between mask conditions [largest *t*(99) = 1.08, *p* = 0.281]; and for Sadness accuracy was higher for the No Mask than either the Posed Mask [*t*(99) = 13.08, *p* < 0.001] or Imposed Mask [*t*(99) = 19.00, *p* < 0.001] conditions, and that the two mask conditions were not significantly different [*t*(99) = 1.76, *p* = 0.082].

Turning to the confidence data, inspection of Fig. 2b (showing mean confidence ratings across the six emotions and three mask conditions) suggests that confidence varied across emotions, was generally higher for the No Mask than the Posed Mask or Imposed Mask conditions, and that the higher confidence for the No Mask condition was present in all emotion conditions other than Anger. These impressions are consistent with the results of the ANOVA analysis with significant effects of emotion [*F*(4.221, 417.9) = 50.165, *p* < 0.001, *η*^2^_*p*_ = 0.336], mask condition [*F*(1.167, 115.5) = 108.68, *p* < 0.001, *η*^2^_*p*_ = 0.523], and an interaction between emotion and mask condition [*F*(8.373, 828.9) = 43.98, *p* < 0.001, *η*^2^_*p*_ = 0.308]. Notwithstanding the mask condition by emotion interaction, follow-up tests of the main effect of mask condition revealed that confidence was generally higher for the No Mask condition than either of the Posed Mask [*t*(99) = 10.83, *p* < 0.001] or Imposed Mask [*t*(99) = 10.57, *p* < 0.001] conditions, and that the two mask conditions were not significantly different [*t*(99) = 0.78, *p* = 0.437].

Given the interaction between emotion and mask condition, follow-up tests were again performed to examine compare the different mask conditions for each emotion separately. These revealed that for Anger there were no significant differences in confidence between mask conditions [largest *t*(99) = 0.65, *p* = 0.520]; for Disgust confidence was higher for the No Mask than either the Posed Mask [*t*(99) = 12.24, *p* < 0.001] or Imposed Mask [*t*(99) = 12.50, *p* < 0.001] conditions, and that the two mask conditions were not significant different [*t*(99) = 1.26, *p* = 0.209]; for Fear confidence was higher for the No Mask than either the Posed mask [*t*(99) = 3.36, *p* = 0.001] or Imposed Mask [*t*(99) = 2.86, *p* = 0.005] conditions, and the two mask conditions not significantly different [*t*(99) = 1.00, *p* = 0.318]; for Happiness confidence was higher for the No Mask than either the Posed mask [*t*(99) = 11.16, *p* < 0.001] or Imposed Mask [*t*(99) = 9.72, *p* < 0.001] conditions, and that the two mask conditions were significantly different [*t*(99) = 2.22, *p* = 0.029]; for Neutral, confidence was higher for the No Mask than either the Posed mask [*t*(99) = 5.88, *p* < 0.001] or Imposed Mask [*t*(99) = 6.34, *p* < 0.001] conditions, and the two mask conditions were significantly different [*t*(99) = 2.05, *p* = 0.043]; and for Sadness confidence was higher for the No Mask than either the Posed mask [*t*(99) = 13.57, *p* < 0.001] or Imposed Mask [*t*(99) = 13.09, *p* < 0.001] conditions, and the two mask conditions were not significantly different [*t*(99) = 1.55, *p* = 0.125].

In summary, these results confirm that the accuracy of emotion recognition from faces is sometimes impaired when the lower part of the face is obscured by masks, but that this effect is not consistent across all emotions tested here. In particular, it was absent or reversed for anger, fear, and neutral expressions, and this is unlikely to be due to ceiling effects because accuracy was highest for happy or sad expressions where there was a clear negative effect of masks. Moreover, while there were some small differences across emotions, accuracy was generally similar for the Posed Mask and Imposed Mask conditions. In terms of the participants confidence in their emotion judgements, this was generally higher for stimuli not obscured by masks (with the exception of angry faces), and as with accuracy, confidence was generally similar between the Posed Mask, and Imposed Mask conditions despite some minor differences across emotions.

## Discussion

The most general observation from the results reported here is that the accuracy of judgements of emotion from facial expressions (and confidence in those judgements) is impaired when the faces being judged were partially obscured by wearing a face mask. However, this high-level summary obscures important aspects of the detail of the results: in particular the fact that the impairment in accuracy was not consistent across emotions (and was reversed in some cases) and that the pattern of effects for accuracy and confidence was different. But before turning to these issues, one of the key motivations for the current study was the possibility that previous investigations of the effects of mask wearing may have been misleading due to their reliance on graphically manipulated stimuli where masks were artificially imposed rather than posed directly. Fortunately, this concern does not seem to have been a material one-both accuracy in emotion detection, and confidence in those judgements, were generally similar for stimuli where the emotions were posed by people wearing masks, and where emotions were posed without masks and masks subsequently added by graphical manipulation. Although there were some minor differences between the Posed Mask and Imposed Mask conditions across emotions, even where present, they were generally small compared to the difference to the No Mask condition. Moreover, it should be remembered that there will be differences in the stimuli created by requiring people to pose emotions multiple times (with/without the mask) that could impact on the ease with which those emotions are judged. Thus, while the use of graphical manipulation remains in principle a potential limitation in studies of this kind, in practice it does not appear to have any materially deleterious effects.

Turning to the observation that impairments in accuracy were not consistent across all emotions tested. This general result is not entirely novel—both Carbon (2020) and Noyes et al. (2021) reported interactions between the effects of mask wearing and emotional expression—with the disruption produced by masks not seen in all emotions. That said, both the specific emotions (fearful or neutral for Carbon; sad and neutral for Noyes et al.) and the explanation offered (ceiling effects for Carbon; specific importance of features in the mouth region for these emotions for Noyes et al.) differed. Our results are consistent with the lack of effect for neutral expressions reported previously (note also that Gulbetekin (2021) reported the smallest effect was for neutral expressions), but also suggested that accuracy was, if anything, higher for anger and fear when judging faces wearing masks. The consistency of effects (or more precisely, lack of effects) for neutral stimuli may reflect prior observations that the eye region is reasonably diagnostic for such (lack of) expression (e.g. Blais et al., 2012; Smith et al., 2005; Wegrzyn et al., 2017) and so may not be expected to be particularly disrupted by occlusion of the mouth region by a mask. It would certainly seem unlikely that ceiling effects (see, Carbon, 2020) could be a general explanation because the neutral expression was recognised with less accuracy in the absence of masks both here and in Carbon (2020) or Noyes et al. (2021) compared to some expressions where there was a mask impairment in accuracy. With respect to the observation that, if anything, accuracy was higher in the presence of masks for anger and fear, it is true that obscuring part of the face may actually improve the accuracy of emotion judgements in at least some circumstances (e.g. Roberson et al., 2012), but such observations are relatively rare. Thus, the current observation of improved detection of anger or fear in the presence of masks may need to be interpreted with some caution in the absence of replication (however, even if this “reversal” of the overall negative effects of masks on these emotions is not reliable, the fact that mask effects are not consistent across emotions would remain, along with the dissociation between this and the consistent effects of mask wearing on confidence). Moreover, while Carbon (2020) reported a lack of impairment produced by masks with fear, Noyes et al. (2021) did not, while both reported impairments for anger (which was not replicated here), and Pazhoohi et al. (2021) report deficits for all expressions examined. In addition, there does not seem to be a clear fit with prior studies of the diagnostic features of the face (e.g. Blais et al., 2012; Smith et al., 2005; Wegrzyn et al., 2017), because they have suggested that the mouth region is typically diagnostic for at least some of the emotions not impaired by masks (but see, Schurgin et al., 2014). Thus, while the fact that mask wearing does not impair all emotions equally is clear across all studies of this issue, there remains uncertainty over which expressions are most (or least) affected. It is also the case that there is little direct match between the effects of mask wearing and prior studies of the diagnostic face features for different emotions. While speculative, one possible account of this heterogeneity of effects across studies is that specific details of the stimuli may have influenced the results. In this light, it is noteworthy that the stimuli from all studies are quite dissimilar: here, photographs posed by young females (not professional actors/models) specifically for the current study; Carbon (2020),^1^ white male and female professional actors/models taken from a wide range of ages in the FACES database (Ebner et al., 2010); Noyes et al. (2021), male and female professional actors/models with a narrow age range across a variety of ethnic backgrounds from the NIMSTIM database (Tottenham et al., 2009); and Pazhoohi et al. (2021), white male and female professional actors/models taken between 19 and 31 years of age also from the FACES database (Ebner et al., 2010).

The other noteworthy aspect of the current results is the discrepancy between the effects of mask wearing on the accuracy of emotion detection and the confidence in those judgements: the participants were generally more confident (anger aside) when viewing faces that were not wearing masks, despite the fact that for some emotions accuracy was either improved by mask wearing or unaffected by it. A similar dissociation we also present (but largely dismissed as an artefact of ceiling effects for accuracy in some emotions) in the results of Carbon (2020), with confidence higher for unmasked faces across all emotions, but accuracy not impaired by masks for all emotions (and similarly, Pazhoohi et al. (2021) report larger effect sizes for mask wearing on confidence than on accuracy). It seems very unlikely that ceiling effects could offer a general account of this dissociation: both here and in Carbon (2020) mask-related accuracy impairments were seen for emotions that were recognised with higher levels of precision (and thus closer to ceiling) than the emotions for which there were no mask-related impairments; and in the current results there were emotions where accuracy was higher with masks than without, which was not seen for confidence judgements in any emotion. Given that this dissociation between accuracy and confidence appears to be both a real and reliable one, perhaps the most general implication is that people appear to over-generalise or over-estimate the potential for masks to impair emotion recognition. Although a discrepancy between accuracy and confidence is in no way unusual in human judgements (perceptual or otherwise), in the current situation it may have material consequences if it leads to mask wearing producing biases in emotion recognition of which people are unaware, or if their lack of confidence (which is some cases appears to be misplaced) interferes with social interactions.

In summary, the current results add to the small literature concerning the effects of mask wearing on emotion recognition: confirming previous observations that while there are impairments for some emotions, this is not true for all; confirming that observers are not particularly accurate in their judgements of when their emotion judgements are likely to be impaired or not; and suggesting that the use of graphically manipulated stimuli is not a material problem for this type of investigation. That said, it is important to note that, even when considered as a whole, this literature does have some potential limitations. Perhaps the most obvious being that the participants used in all studies came from relatively restricted and primarily WEIRD groups (Henrich et al., 2010). Given that mask wearing is relatively novel in general society for the countries in which the participants in these studies lived (Canada, Italy, Germany, Turkey, the UK, and the USA) and the idea that emotion recognition is a cultural universal has been questioned (e.g. Jack et al., 2012), then investigating whether the effects observed thus far generalise to other cultures will be particularly important. This is partially a general issue of the potential differences between cultures in facial emotion recognition, but also one relating to the fact that facemask wearing has been relatively common in some societies (in particular within Asia) prior to the current pandemic and the additional familiarity may affect the degree or manner in which mask wearing affects emotion recognition. In addition, the fact that the list of emotions that are exceptions to the general rule of impairment produced by mask wearing differs across studies, and the possibility this may be due to differences in the nature of the face images being judged, implies that further work is needed to fully establish when masks will (and will not) interfere with emotion recognition. Unfortunately, the fact that the COVID-19 pandemic appears far from over suggests that there may be more than sufficient opportunity to resolve these unanswered questions before mask wearing is no longer a valuable public health measure.

## Supplementary Information

**Additional file 1**. Re-analysis including only participants self-reported to be female and white ethnicity.

## Data Availability

The datasets used during the current study are available from the corresponding author on reasonable request.

## Footnotes

1 One implication of using images posed by non-professional models in the current study is that these have not received external validation for the expressions displayed. It is clearly possible that variation between emotions (and between the No Mask and Posed Mask conditions) in terms of the clarity of the emotional displays may have affected the results. However, the fact that all emotions were well-recognised in the No Mask condition would suggest the posed expressions were at least reasonably clear. Moreover, the fact that the effects of Posed and Imposed masks were similar, despite the Imposed Mask stimuli being created from the No Mask images, would suggest that differences in the clarity of emotional expression between mask conditions is unlikely to be driving the results presented here.
