# Supplementary material for: Face masks have emotion-dependent dissociable effects on accuracy and confidence in identifying facial expressions of emotion
Source: Cogn Res Princ Implic. 2022 Feb 14;7:15. doi: 10.1186/s41235-022-00366-w (PMC8844328; doi:10.1186/s41235-022-00366-w)
Supplement: Supplementary file 1 — Additional file 1. Re-analysis including only participants self-reported to be female and white ethnicity. [file 41235_2022_366_MOESM1_ESM.docx]

Additional Analysis For

Face masks have emotion-dependent dissociable effects on accuracy and confidence in identifying facial expressions of emotion.

Emily Grenville and Dominic M. Dwyer

(School of Psychology, Cardiff University)

Short title: Face masks & emotion recognition

*Cognitive Research: Principles and Implications*

(Article collection on “Face Coverings: Considering the implications for face perception and speech communication”)

Address for correspondence:

Dominic M. Dwyer, School of Psychology, Cardiff University, Tower Building, Park Place, Cardiff, CF10 3AT, UK; Tel: +44 (0)29 20876285; Email: DwyerDM@cardiff.ac.uk

**Description/Summary**

The presentation of the results in the main paper was based on all 100 subjects tested. However, the sample was unevenly distributed in terms of the self-reported gender and ethnicity of the subjects, with the largest subset (N = 74) female and of self-reported white ethnicity. This Additional Analysis File reports a full analysis (performed exactly as in the main paper) of only this largest subset of the subjects. Except as where explicitly noted, the analysis of the full subjects set and this restricted subset are entirely congruent.

**Results**

Inspection of Additional Figure 1A (showing mean percentage correct emotion identification across the six emotions and three mask conditions) suggests that overall accuracy varied across emotions, was generally better for the No Mask than the Posed Mask or Imposed Mask conditions, but that the effect of mask condition was not consistent across emotions (in particular, the advantage for the No Mask condition appears negligible or reversed for Anger, Fear, and Neutral emotions). These impressions are consistent with the results of the ANOVA analysis with significant effects of emotion [F(3.538, 258.69) = 53.00, p < 0.001, η^2^_p_ = 0.421], mask condition [F(2, 146) = 120.01, p < 0.001, η^2^_p_ = 0.622], and an interaction between emotion and mask condition [F(7.332, 535.27) = 57.44, p < 0.001, η^2^_p_ = 0.440]. Notwithstanding the mask condition by emotion interaction, it is potentially informative that follow up tests of the main effect of mask condition revealed that accuracy was generally higher for the No Mask condition than either of the Posed Mask [t(73) = 13.23, p < 0.001] or Imposed Mask [t(73) = 12.72, p < 0.001] conditions, and that the two mask conditions were not significantly different from each other [t(73) = 1.34, p = 0.184].

Given the interaction between emotion and mask condition, follow-up tests were performed to compare the different mask conditions for each emotion separately. These revealed that for Anger accuracy was lower for the No Mask than either the Posed mask [t(73) = 2.06, p = 0.043] or Imposed Mask [t(73) = 4.71, p < 0.001] conditions, and that the two mask conditions were themselves significantly different [t(73) = 2.99, p = 0.004]; for Disgust accuracy was higher for the No Mask than either the Posed mask [t(73) = 13.72, p < 0.001] or Imposed Mask [t(73) = 15.47, p < 0.001] conditions, and that the two mask conditions were not significantly different [t(73) = 0.48, p = 0.632]; for Fear accuracy in the No Mask was not significantly different to the Posed mask condition [t(73) = 0.45, p = 0.654], but was lower than the Imposed Mask [t(73) = 2.40, p = 0.019] condition, and the two mask conditions were themselves significantly different [t(73) = 3.25, p = 0.002]; for Happiness accuracy was higher for the No Mask than either the Posed Mask [t(73) = 4.83, p < 0.001] or Imposed Mask [t(73) = 3.74, p < 0.001] conditions, and that the two mask conditions were not significantly different [t(73) = 0.76, p = 0.453]; for Neutral, there were no significant differences in accuracy between mask conditions [largest t(73) = 0.73, p = 0.470]; and for Sadness accuracy was higher for the No Mask than either the Posed Mask [t(73) = 10.81, p < 0.001] or Imposed Mask [t(73) = 16.22, p < 0.001] conditions, and that the two mask conditions were not significantly different [t(99) = 1.52, p = 0.131].

Turning to the confidence data, inspection of Additional Figure 1B (showing mean confidence ratings across the six emotions and three mask conditions) suggests that confidence varied across emotions, was generally higher for the No Mask than the Posed Mask or Imposed Mask conditions, and that the higher confidence for the No Mask condition was present in all emotion conditions other than Anger. These impressions are consistent with the results of the ANOVA analysis with significant effects of emotion [F(4.006, 292.41) = 36.58, p < 0.001, η^2^_p_ = 0.334], mask condition [F(1.166, 85.11) = 78.55, p < 0.001, η^2^_p_ = 0.518], and an interaction between emotion and mask condition [F(8.154, 595.28) = 35.06, p < 0.001, η^2^_p_ = 0.324]. Notwithstanding the mask condition by emotion interaction, follow up tests of the main effect of mask condition revealed that confidence was generally higher for the No Mask condition than either of the Posed Mask [t(73) = 9.42, p < 0.001] or Imposed Mask [t(73) = 9.06, p < 0.001] conditions, and that the two mask conditions were not significantly different [t(73) = 0.56, p = 0.577].

Given the interaction between emotion and mask condition, follow-up tests were again performed to examine compare the different mask conditions for each emotion separately. These revealed that for Anger there were no significant differences in confidence between mask conditions [largest t(73) = 0.72, p = 0.469]; for Disgust confidence was higher for the No Mask than either the Posed Mask [t(73) = 11.40, p < 0.001] or Imposed Mask [t(73) = 11.30, p < 0.001] conditions, and that the two mask conditions were not significant different [t(73) = 0.97, p = 0.337]; for Fear confidence was higher for the No Mask than either the Posed mask [t(99) = 2.87, p = 0.005] or Imposed Mask [t(73) = 2.31, p = 0.024] conditions, and the two mask conditions not significantly different [t(73) = 0.76, p = 0.449]; for Happiness confidence was higher for the No Mask than either the Posed mask [t(73) = 10.08, p < 0.001] or Imposed Mask [t(73) = 9.01, p < 0.001] conditions, and that the two mask conditions were not significantly different [t(73) = 1.18, p = 0.241: note, this was different to the main analysis of all 100 subjects, where this comparison did reveal a significant difference]; for Neutral, confidence was higher for the No Mask than either the Posed mask [t(73) = 4.78, p < 0.001] or Imposed Mask [t(73) = 5.01, p < 0.001] conditions, and the two mask conditions were not significantly different [t(73) = 1.69, p = 0.096: note, this was different to the main analysis of all 100 subjects, where this comparison did reveal a significant difference]; and for Sadness confidence was higher for the No Mask than either the Posed mask [t(73) = 11.71, p < 0.001] or Imposed Mask [t(73) = 10.68, p < 0.001] conditions, and the two mask conditions were not significantly different [t(99) = 1.70, p = 0.092].

In summary, with the (very) minor exception of the lack of a difference in confidence between the Posed and Imposed mask conditions for Happy and Neutral faces, the analysis reported in the main paper for all 100 subjects is entirely congruent with that for the subset of subjects self-reporting as female and of white ethnicity.

**Additional Figure 1**


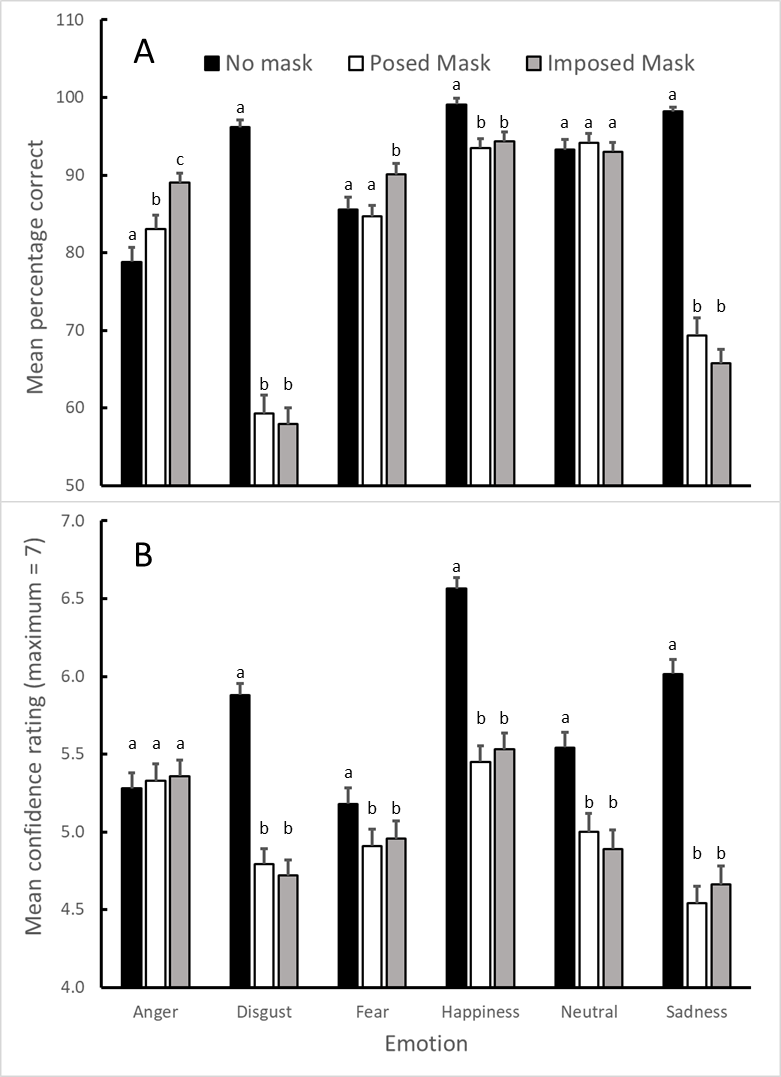


Shows, (A) Mean percentage correct (with SEM) identification of emotional state, and (B) mean confidence rating (with SEM), as a function of emotion and mask condition. Note – superscript letters indicate the presence/absence of significant differences between mask conditions for each emotion: conditions with different letters are significantly different from each other (p < 0.05), conditions with the same letter are not significantly different (p > 0.05).
